# Monoclonal antibodies for migraine: an update

**DOI:** 10.1007/s00415-018-8886-8

**Published:** 2018-05-14

**Authors:** Daniel Castle, Neil P. Robertson

**Affiliations:** Department of Neurology, Institute of Psychological Medicine and Clinical Neurosciences, Cardiff University, Cardiff, CF14 4XW UK

## Introduction

Migraine is an episodic neurological disorder resulting in attacks of headache associated with neurological symptoms. Whilst episodes of migraine are debilitating for the patient, they also result in a significant burden for society as a whole. The World Health Organisation now ranks migraine as the third most prevalent medical condition in the world, and the second most disabling neurological condition. The economic cost of migraine is also considerable and has an estimated worldwide annual cost of $20 billion, relating to healthcare, procedures and loss of productivity.

A number of therapeutic avenues have been explored to combat migraines over the decades, with perhaps the most significant milestone occurring in the 1990s with the introduction of serotonin 5-HT1B/1D receptor agonists which led to improvement in the management of acute migraine, albeit with some continuing concerns regarding cardiovascular side effects. However, despite the widespread use of these medications, only a third of patients have sustained freedom from pain.

The relevance of calcitonin gene-related peptides (CGRP) in the pathophysiology of migraine was first identified in 1990 by Goadsby et al. who established that CGRP levels were increased in the cranial venous outflow during acute genuine migraine attacks. Further studies demonstrated that those treated successfully with triptans during a migraine attack had a drop in their level of CGRP. Furthermore, peripheral infusion of CGRP induced an attack in those known to suffer with migraine but appeared to have little effect on healthy volunteers. As a result, a range of monoclonal antibodies have been produced which target the peptide itself or its corresponding receptor.

This month’s journal club will review clinical trial results for a series of novel monoclonal antibodies that target CGRP in patients with migraine. The first paper examines the use of erenumab in the STRIVE study for episodic migraine; the second paper updates our understanding of fremanezumab for chronic migraine and finally galcanezumab in episodic migraine.

## A controlled trial of erenumab for episodic migraine

In this well publicised international multicentre, randomised, double-blind, placebo-controlled, parallel-group phase three trial, Goadsby et al. investigated the efficacy of the drug erenumab in treating episodic migraine. Erenumab is a monoclonal antibody directed against the canonical CGRP receptor. In this study, 955 patients were randomly allocated to receive a subcutaneous injection of erenumab 70 mg, erenumab 140 mg or placebo once a month for 6 months. Participants were assessed over a 4-week period to ensure they met trial inclusion criteria as well as establishing a baseline data set. This included data on migraine frequency, medication use and functional impact of migraine as measured through a daily electronic diary of migraine and headache symptoms.

The study demonstrated a statistically significant fall in migraine days from baseline assessment, when adjusted for placebo, of 1.4 days in the 70 mg group and 1.9 in the 140 mg group. There was also a 50% reduction in migraine days when adjusted for placebo seen in 23.4% of patients in the 140 mg cohort and 16.7% in the 70 mg. Secondary outcomes included statistically significant reductions compared to placebo in use of acute migraine-specific medications and patient functional abilities as assessed with migraine physical function impact diary. Similar rates of adverse incidents were observed across all 3 cohorts with mild to moderate needle site injection reactions being most common. No cardiac or liver abnormalities were observed but 35 out of 628 post baseline antibody tests were positive to anti-erenumab antibodies with one patient in the 70 mg group developing neutralising antibodies.

*Comments*: This well-powered and designed study has demonstrated the potential benefits of erenumab in episodic migraine management. However, as noted by the authors, a limitation of this study was the exclusion of patients who had had a lack of therapeutic response to more than two classes of migraine preventative drugs as well as women of childbearing potential. Inclusion of these patient groups clearly offers challenges, but since refractory females of childbearing age may be a highly relevant target group for this drug, the additional complexities may have been worth it.

Goadsby et al. (2017) New England Journal of Medicine 30;377(22):2123–2132

## Fremanezumab for the preventive treatment of chronic migraine

This paper by Silberstein et al. examines treatment of chronic migraine with fremanezumab, an antibody that binds to CGRP itself. This double-blind, randomised, placebo-controlled phase 3 trial assessed 3148 participants over a 4-week baseline period, recruiting 1130 eligible patients. Participants were divided into a placebo, monthly and quarterly group. Each group received a subcutaneous injection once a month, with the monthly group receiving fremanezumab every month and the quarterly group receiving fremanezumab on the first injection and placebo thereafter. Functional outcomes were assessed with an electronic diary and headache impact test questionnaire. The primary outcome was reduction of headache days compared with baseline. A headache day was defined as any day in which head pain lasted ≥ 4 consecutive hours with a peak severity of at least moderate, or the use of acute migraine medications. Secondary outcomes included drug safety profiling, reduction in migraine days and percentage reduction by 50%.

Fremanezumab demonstrated statistically significant improvement when adjusted for placebo and reduced headache days by 1.8 days in the quarterly group and 2.1 days in the monthly group. Reduction in migraine days was also significant, with 1.7 in the quarterly and 1.8 days reduction in the monthly group, when adjusted for placebo. Mild to moderate needle site reactions were once again the most common adverse event. There was some evidence of transient liver function test derangement, but may have been related to concurrent medications. One participant on fremanezumab developed suicidal ideation leading to trial discontinuation.

*Comments*: The statistically significant treatment effect for headaches lasting over 4 h and to a lesser extent migraine is promising. However, the primary objective in this trial was reasonably vague and covered treatment of migraine and non-migrainous head pain, potentially complicating its indication. However, the study does suggest that fremanezumab is effective and also offers a unique and attractive treatment regimen of four times per year administration.

Silberstein et al (2017) New England Journal of Medicine 30;377(22):2113–2122

## Effect of different doses of galcanezumab vs placebo for episodic migraine prevention a randomised clinical trial

The final paper is a phase 2b placebo controlled, dose-ranging study of galcanezumab (which selectively binds CRGP) in episodic migraine. 936 patients were reviewed over a screening and baseline period and 410 patients enrolled. Patients were randomised to monthly; placebo, 5, 50, 120 and 300 mg intervention groups in a 2:1:1:1:1 order, respectively. Patients received a subcutaneous injection once a month for 3 months with drug or placebo. Functional outcomes were assessed by reporting to an automated phone line and completion of the headache impact test questionnaire.

The primary objective was to assess whether at least one dose of galcanezumab was superior to placebo in the prevention of migraine, by reduction in migraine headache days (MHDs). MHD was defined as any calendar day in which a migraine headache lasted ≥ 30 min, the use of acute migraine treatment was not automatically counted as an MHD. After placebo adjustment, the 120 mg monthly cohort demonstrated the only statistically significant response, with a reduction in MHD of 1.1. Safety profiling demonstrated relatively good outcomes. Mild or moderate injection site reactions were common. One participant was diagnosed with Crohn’s disease, another treated for appendicitis and one developed suicidal ideation, which the investigators felt were unrelated to the drug.

*Comments*: The level of MHD reduction observed, and proportion with 50% reduction of migraine days is comparable if not inferior to some current therapies including topiramate, propranolol and acupuncture. As a result, an analysis of cost effectiveness is likely to be relevant and trials which consider these drugs against an active comparator would be enlightening. Although the safety profile was generally good, the participants in this and the previous study who developed suicidal ideation may be a feature to monitor going forward.

Skljaarevski et al (2018) JAMA Neurology 75(2):187–193

